# The AMP-Activated Protein Kinase (AMPK) Positively Regulates Lysine Biosynthesis Induced by Citric Acid in *Flammulina filiformis*

**DOI:** 10.3390/jof9030340

**Published:** 2023-03-10

**Authors:** Hao Fan, Feng Ge, Tao Wu, Yongzhi Liu, Li Tian, Yueqian Liu, Taobo Xiang, Hanshou Yu, Liang Shi, Qin He, Ang Ren, Ailiang Jiang

**Affiliations:** Sanya Institute of Nanjing Agricultural University, Key Laboratory of Agricultural Environmental Microbiology Ministry of Agriculture, Department of Microbiology, College of Life Sciences, Nanjing Agricultural University, Nanjing 210095, China

**Keywords:** *Flammulina filiformis*, lysine biosynthesis, AMPK, inducing factor, citric acid, response surface methodology

## Abstract

*Flammulina filiformis*, the most produced edible mushroom species in China, is rich in lysine. Further enhancing its lysine biosynthesis is vital for improving its quality in industrialized cultivation. Citric acid induction significantly increases both the biomass and growth rate of *F. filiformis* hyphae, as well as the lysine content. The genes encoding enzymes in the lysine biosynthesis pathway were detected under the optimal induction, revealing that the expression levels of *hcs*, *hac*, and *hah* were 2.67, 1.97, and 1.90 times greater, respectively, relative to the control, whereas no significant difference was seen for *hdh*, *aat*, *sr*, and *shd*, and the expression of *aar* decreased. Furthermore, the transcriptional levels of *Ampk*, *GCN2*, *GCN4*, and *TOR* were found significantly upregulated, with the most upregulated, *Ampk*, reaching a level 42.68 times greater than that of the control, while the phosphorylation of AMPK rose by nearly 54%. In AMPK-silencing strains under the optimal induction, however, the phosphorylation increment dropped to about 16% and the lysine content remained at the same level as in the WT. Thus, AMPK is presented as the critical intermediary in citric acid’s regulation of lysine biosynthesis in *F. filiformis*.

## 1. Introduction

*Flammulina filiformis* (Z.W. Ge et al. P.M. Wang et al.) [1], also known as golden needle mushroom or winter mushroom, is one of the most produced and consumed edible mushroom species in China [2,3]. It is also a species of great interest in macrofungus research today [4,5]. With the gradual application of industrialized large-scale production of *F*. *filiformis*, annual production has reached around 100,000 tons. Nowadays, China has become the world’s largest producer of *F*. *filiformis*. Although the production of *F*. *filiformis* is increasing year after year, its quality development has stagnated for a long time. Thus, the future of *F*. *filiformis* factory production needs to transfer its focus from expanding production scale and increasing yields to improving quality [6]. *F. filiformis* is broadly appreciated by customers for being rich in fiber, low in fat and energy, and containing abundant essential amino acids and vitamins [7], especially its rich content of lysine, which is higher than in other edible mushroom species [8]. Lysine helps improve mental development, specifically among children, hence the name “intelligence-enhancing mushroom”. Therefore, it has become an important development direction to improve the quality of *F*. *filiformis* by further enhancing its lysine biosynthesis in industrialized large-scale production.

To meet consumers’ rising demand for food quality, the production mode must be updated [9]. Existing studies tend to focus on enhancing biosynthetic capacity through genetic engineering, which invariably requires a great deal of time and effort and is hard to introduce into large-scale factory production. The mode of modern factory production of edible fungi provides a standardized model for growth regulation, which allows the adjustment of process parameters [10,11]. Regulating cultivation parameters to further increase lysine content during the production process is an important way to improve the quality of *F. filiformis*. Environmental parameters determine the yield and quality of edible mushrooms during their growth. Existing studies have applied several stimulation factors during cultivation to promote the growth of the fruiting body of edible mushrooms or the accumulation of secondary metabolites, such as cold stimulation of *Pleurotus tuoliensis* (C.J. Mou M.R. Zhao and Jin X. Zhang) mature mycelia [12], which was able to induce the formation of fruiting bodies and which also played an important role in increasing yields [13]. Exogenous Na^+^ and Mn^2+^ addition was able to enhance GA production in *Ganoderma lucidum* [14,15]. In our previous work, chitosan, as a homologue of the fungal cell wall component chitin, was shown to significantly increase the lysine content of *F. filiformis* as well as mycelium growth, and the amino acid transporter (AAT) was further revealed to be involved in the regulation mechanism [16]. That study not only provided a method to increase the lysine content of *F. filiformis* in factory production, but also revealed the significant role AATs play in the regulation of the quality of *F. filiformis*. The result is on track to be applied in large-scale industrial trials which will provide new ideas for *F. filiformis* quality optimization. However, the focus was limited to the amino acid transportation process into and out of cells and organelles, rather than the lysine biosynthesis pathway and its regulation. Considering the significant increase in growth and lysine content, the efficiency of lysine biosynthesis and regulatory mechanism of *F. filiformis* still needed to be further investigated.

Lysine is an amino acid that cannot be synthesized by monogastric animals and is unable to participate in transamination, so it must be obtained from food to meet the need for growth and metabolism. Although cereals contain small amounts of lysine, it is vulnerable to processing and the amounts are insufficient for daily life. However, lysine is present in higher concentrations in certain bacteria and fungi, such as *F. filiformis* [17]. Lysine biosynthesis in *F. filiformis* is conducted through the α-aminoadipate (AAA) pathway [18]. α-ketoglutarate acts as the precursor substance which goes through an eight-step enzyme-catalyzed reaction involving seven free intermediates. First, α-ketoglutarate is catalyzed by homocitrate synthase (HCS) with the participation of acetyl-CoA to form homocitrate, then catalyzed by homoaconitase (HAC) to form *cis*-homoaconitate. Next, *cis*-homoaconitate is catalyzed by homoaconitate hydratase (HAH) to form homoisocitrate, followed by its catalyzation by homoisocitrate dehydrogenase (HDH) with the participation of NAD^+^ forming α-ketoadipate. Then, α-aminoadipate is formed from α-ketoadipate and glutamate by the action of aminoadipate aminotransferase (AAT), which is followed by aminoadipate reductase (AAR), saccharopine reductase (SR) and saccharopine dehydrogenase (SDH) to complete the final step of lysine biosynthesis. The first half of the α-aminoadipate pathway takes place in the mitochondria; this is followed by the biosynthesis of homocitrate in the nucleus, and the last step of converting α-aminoadipate to lysine occurs in the cytoplasm. The first half of the α-aminoadipate pathway has many similarities to the tricarboxylic acid (TCA) cycle, with the early lysine biosynthesis precursors merely one carbon higher homologue. Thus, most enzymology of the α-aminoadipate pathway is very similar to that found in the TCA cycle [18]. The two critical enzymes, homoaconitase (HAC) and homoaconitate hydratase (HAH), are all located in the mitochondria and contain mitochondrial substances such as mitochondrial transfer peptide, indicating that lysine biosynthesis is closely associated with energy metabolism. Organic acid metabolism is of great significance at the cellular level in several biochemical pathways, including energy production, the formation of amino acid biosynthetic precursors, and regulation of the level of plant adaptation to the environment [19]. It has been found that the exogenous addition of citric acid shortened the postripening period and increased the fruiting body yield of *Hypsizygus marmoreus* by enhancing the activity of the TCA cycle [20]. Citric acid promotes growth, is involved in energy metabolism as an intermediate metabolite in the TCA cycle, and also acts as a precursor for lysine biosynthesis, implying a potentially important role in the improvement in quality of *F. filiformis*.

In this work, we demonstrated a possible way to improve the lysine content of *F. filiformis* in industrialized large-scale production. Citric acid was applied as the inducer to enhance the production of lysine. The expression level of critical enzymes and regulation factors in lysine biosynthesis was detected in order to analyze the molecular mechanism of citric acid regulation on lysine biosynthesis. This technique promises to become one of the important directions for the future improvement in the quality of *F. filiformis* in factory production.

## 2. Experimental Procedures

### 2.1. Strains and Culture Conditions

The *F. filiformis* strain Fv3 from the Shanghai Academy of Agricultural Sciences was inoculated on PDA solid medium and cultured at 25 °C for 5 days. The activation plate was then pierced with a punch and eight colonies were inoculated into 250 mL shake flasks containing 100 mL of liquid PDA medium with an inoculating needle. The resulting mycelia were incubated at 25 °C and 150 rpm for 5 days, then aseptically disrupted, and 3 mL was inoculated into shake flasks containing 100 mL of PDA. Each was incubated at 150 rpm and 25 °C for 5 days.

### 2.2. Mycelium Treatment Conditions

After 3 days of culture, different concentrations of citric acid were added, and the same amount of sterile water (100 μL) was added to the control group, and this was repeated three times for each group. The concentrations of citric acid were 0 μM, 100 μM, 200 μM, 300 μM, and 400 μM. The effects of citric acid on the lysine and protein content of *F. filiformis* were determined in liquid medium.

### 2.3. Fruiting Body Cultivation of F. filiformis Induced by Citric Acid

The cultivation of fruiting bodies was performed according to previously reported methods with appropriate modifications [16,21]. Citric acid was dissolved in deionized water, then sprayed on the surface of *F. filiformis* culture material after removing the old hyphae. Deionized water was used as control. When the primordia were fully differentiated and the cap was formed, the temperature was lowered to 4–8 °C. When the fruiting bodies were about 2 cm from the mouth of the vase, the temperature was controlled around 5 °C and the fruiting bodies were bagged for inhibition. The fruiting bodies were harvested when the stems reached a certain length.

### 2.4. Measurement of Mycelium Biomass and Growth Rate

The mycelium after fermentation was collected and dried at 60 °C. The weight of the dried mycelium was taken as the mycelial biomass. Different strains with uniform growth were drilled and inoculated into CYM culture medium with patches of the same size. Each strain was inoculated into three different plates, and the colony diameter was measured every 24 h until the mycelia covered a 5 cm plate. Each plate was inoculated with one patch, and each strain was in triplicate. After 3–5 days of growth, photos were taken for observation.

### 2.5. Determination of Lysine Content

The lysine content was measured as described previously [16]. A ninhydrin spectrophotometer was used to determine the lysine content in *F. filiformis*.

### 2.6. Optimization by Response Surface Methodology

The treatment time and concentration of citric acid were selected as independent variables (X) and lysine content as dependent variable (Y). A central composite design (CCD) was used to determine the optimal conditions for the lysine content of *F. filiformis* induced by citric acid. Subsequent experiments were carried out according to the optimal conditions to obtain the actual lysine content of *F. filiformis*, and the difference between the actual lysine content and the predicted lysine content was analyzed.

### 2.7. RNA Extraction and Gene Expression Analysis

The method of total RNA extraction and gene expression analysis was performed as described previously [22,23]. The primers used for Q-PCR can be found in Table S4.

### 2.8. AMPK-Silencing Strain Construction

The silenced transformants were constructed using an RNA interference (RNAi)-mediated gene silencing strategy. The fungal RNAi vector pAN7-dual was used for the construction of AMPK-silencing strains of *F. filiformis*. Fragments of the *Ampk* coding regions were amplified by PCR using *F. filiformis* cDNA as the template and the primers LYZ-*AMPK*i-F: ACTGGGTACCGCGACAAGACGAAACCG and LYZ-*AMPK*i-R: ACTGACTAGTGGCCACCACGTATAACCT. The amplified DNA fragments were cloned into pMD19-T replicative vector and then sent for sequencing. The amplified DNA fragments were subsequently cloned into the pAN7-ura3-dual vector and transformed into *F. filiformis*. The transformants were selected on CYM medium containing 8 μg/mL hygromycin B at 25 °C for 6 days. The silencing transformants were selected and examined with Q-RT PCR to check the gene expression level.

### 2.9. Western Blot for AMPK Total Protein and Phosphorylation Levels

The method of Western blotting was performed as described previously [24].

### 2.10. Statistical Analysis

The experimental data from at least three independent repeats were analyzed using Duncan’s multiple range test and plotted using GraphPad Prism 8. Significant differences in the graph were represented by different letters between the different treatments. *p* < 0.05 was considered to be significant.

## 3. Results

### 3.1. Citric Acid Induction Increased the Biomass, Growth Rate, and Lysine Content of F. filiformis

**Figure 1:**
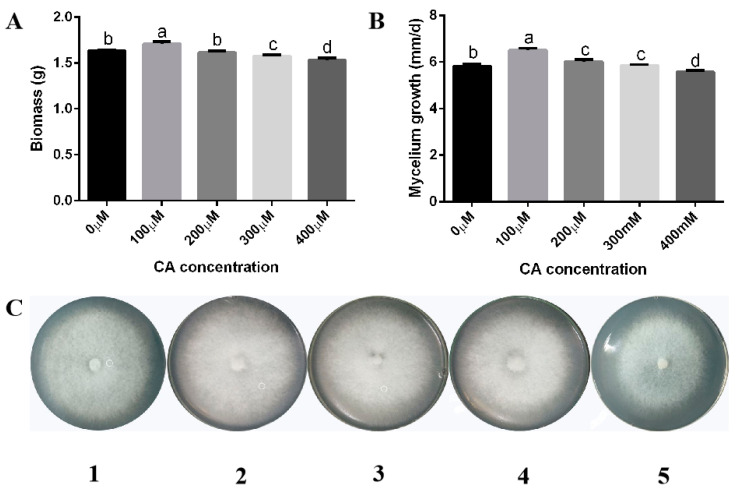
Effects of citric acid on biomass and growth rate of *Flammulina filiformis* mycelia. (**A**) The content of biomass induced by different concentrations of citric acid was detected in WT strain of *F. filiformis*. Different letters indicate a significant difference between treatment groups (*p* < 0.05). (**B**) A statistical graph of the growth rate of the flat hyphae. Different letters indicate a significant difference between treatment groups (*p* < 0.05). (**C**) 1–5 represent the growth state of *F. filiformis* flat hyphae after addition of citric acid content of 0, 100, 200, 300, and 400 μM, respectively.

**Figure 2:**
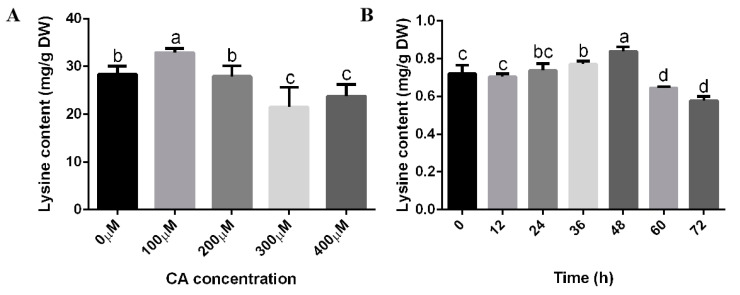
Effects of citric acid and treatment time on lysine and protein in *Flammulina filiformis* mycelium. (**A**) The content of lysine induced by different concentrations of citric acid was detected in WT strain of *F. filiformis*. Different letters indicate a significant difference between treatment groups (*p* < 0.05). (**B**) The content of lysine induced by 100 μM citric acid was detected in WT strain of *F. filiformis*. Different letters indicate a significant difference between treatment groups (*p* < 0.05).

In order to study the effect of citric acid on lysine biosynthesis in *F. filiformis*, different concentrations of citric acid were added for induction. The results can be seen in Figure 1, where 100 μM citric acid increased the biomass of *F. filiformis* mycelium by 4.68% compared with the control (Figure 1A). When the concentration of citric acid was higher than 300 μM, the biomass of *F. filiformis* mycelium was slightly reduced compared with the control. Moreover, the growth rate of *F. filiformis* hyphae was increased by 5.53% compared with the control after 100 μM citric acid treatment (Figure 1B,C). The content of lysine in the mycelium of *F. filiformis* increased first and then decreased, and the lysine content increased by 10.20% under the treatment of 100 μM citric acid (Figure 2A). The above results showed that the growth rate of *F. filiformis* hyphae, the biomass, and the content of lysine in *F. filiformis* mycelium were significantly increased under 100 μM citric acid treatment. Furthermore, under the induction condition of 100 μM citric acid, different induction times also had different effects on the accumulation of lysine in *F. filiformis*. After 100 μM citric acid induction for 24, 36, and 48 h, lysine content increased by 2.52%, 7.11%, and 12.87%, respectively (Figure 2B). The maximum increase was observed after 48 h induction, and the biosynthesis of lysine was significantly inhibited after 60 h induction.

### 3.2. Response Surface Methodology to Optimize Citric Acid Induction Conditions

The CCD test design is shown in Table S2, and the response surface model analysis and the regression analysis are shown in Table S3. The quadratic multiple regression equation obtained after software analysis was Content = 33.78 − 0.042 × A + 0.085 × B + 0.29 × A × B − 4.88 × A^2^ − 5.02 × B^2^.

**Figure 3:**
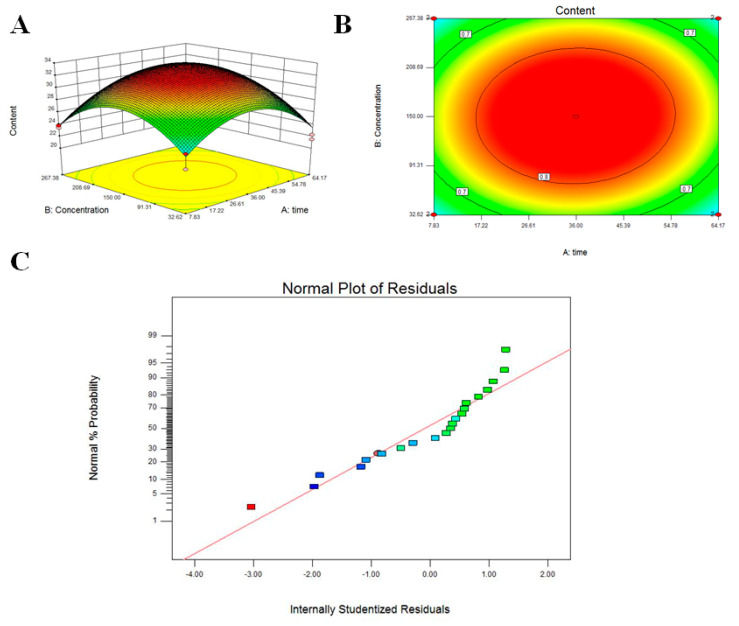
Results of response surface methodology for the optimization of lysine content induced by citric acid in *Flammulina filiformis*. (**A**) 3D response surface view. A 5-level 2-factor design requiring 21 experimental combinations was employed in this optimization study. Table S2 shows the range and level of the independent variables. Each experiment was repeated three times and the mean lysine value was calculated. The experimental data were analyzed by a second-order polynomial regression. (**B**) Contour plots reflect the combined effects of concentration and treatment time on lysine content in *F. filiformis*. (**C**) Residual normal probability distribution map. Each color block is a response value.

As shown in Table S3, the model’s *p*-value of <0.0001 indicated that the regression equation of the model was very significantly different. The *p*-value of 0.8847 > 0.05 for the primary term was not significant, indicating that the lysine content was not simply linear with time; the *p*-value of <0.0001 for the secondary term was highly significant, indicating that the lysine was curved to increase; and the F-value of 16.02 for the misfit term after ANOVA indicated that the three-factor secondary term of the equation had an extremely significant effect on the increase in lysine content in *F. filiformis*. The treatment response surface methodology revealed the optimal citric acid induction time and concentration to be 33.18 h and 138.26 μM and the obtained theoretical lysine content to be 33.92 mg/g DW, with the actual measurements obtained being 32.4378, 33.45 and 33.78 mg/g DW, which were in line with the theoretical results (Figure 3).

### 3.3. Citric Acid Induction Changes the Lysine Content of F. filiformis Fruiting Bodies

**Figure 4:**
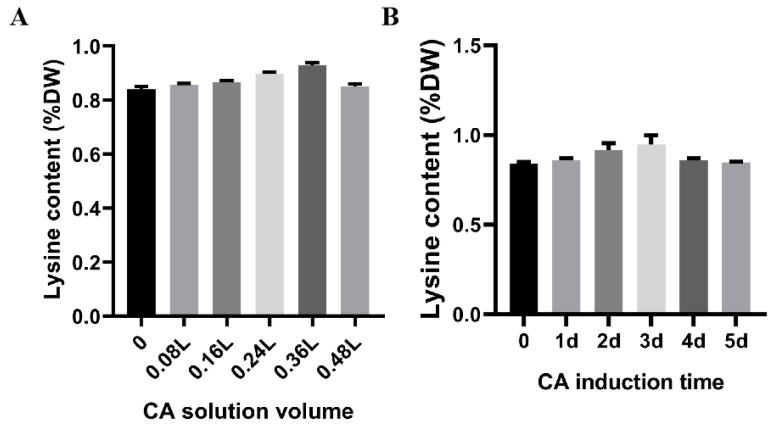
Effects of different citric acid inductions on the content of lysine in fruiting body of *Flammulina filiformis*. (**A**) Effect of different contents of citric acid on the content of lysine in fruiting body. (**B**) Effect of citric acid induction time on lysine content in fruiting body.

Sterile water was used in the control group, and 0, 0.08 L, 0.16 L, 0.24 L, 0.32 L, and 0.48 L of 1 mM citric acid were sprayed for 2 days at the stage during which they needed to be watered during cultivation. The lysine content in the fruiting body increased to varying degrees after two days of induction, with 0.36 L citric acid inducing the highest lysine content of 10.36% compared to the control (Figure 4A). Then, 1–7 days of induction using 0.36 L of 1 mM citric acid increased the lysine content in the fruiting body of *Flammulina filiformis*, with the highest lysine content being 12.86% more than the control after 3 days of induction (Figure 4B). Therefore, citric acid is able to promote lysine biosynthesis in *F. filiformis* fruiting bodies.

### 3.4. Changes in Lysine Content, Gene Transcription Level, and Key Regulators under Optimal Citric Acid Induction Conditions

**Figure 5:**
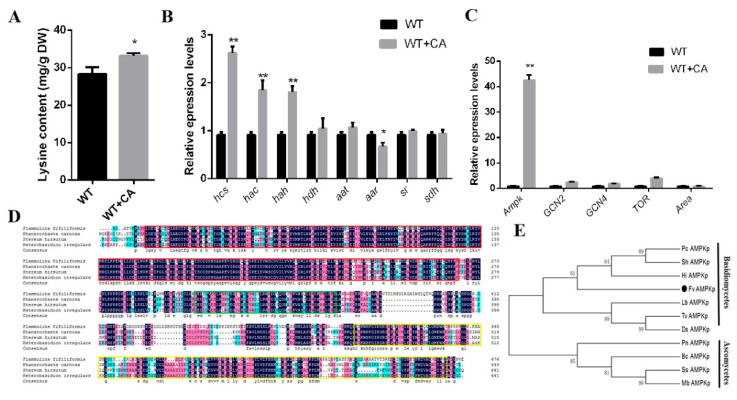
Molecular mechanism of citric acid regulation of lysine biosynthesis in *Flammulina filiformis* as well as phylogenetic analysis and alignment of the AMPK domains. The asterisks indicate significant differences compared with the untreated strains (Student’s *t*-test: * *p* < 0.05 and ** *p* < 0.01). (**A**) Lysine content of *F. filiformis* under optimal citric acid induction. (**B**) Changes in transcriptional levels of lysine-synthesis-related enzymes induced by citric acid. Error analysis represents the standard deviation of the three experimental replicates for each treatment. (**C**) Changes in transcriptional levels of key regulators of amino acid metabolism and energy metabolism under optimal conditions of citric acid induction. (**D**) Alignment of AMPK protein sequences of *F. filiformis* with other basidiomycetes. The dark blue shaded part is 100% conserved; the pink shaded part is 75% conserved; the light blue shaded part is 50% conserved. The red box is conserved protein kinase domain structure; the green box is ubiquitin-associated (UBA) domain structure; the yellow box is AMPK C-terminal α-subunit regulatory structure. (**E**) Phylogenetic tree analysis of AMPK protein of *F. filiformis* and homologous protein of AMPK in fungi. Sequence alignment and construction of phylogenetic trees of AMPK proteins in fungi were performed using MEGA.6.0. Sequence of other fungal AMPK homologous proteins used to construct phylogenetic trees: *Phanerochaete carnosa* (Pc AMPKp, XP_007391270.1), *Stereum hirsutum* (Sh AMPKp, XP_007299169.1), *Heterobasidion irregulare* (Hi AMPKp, XP_009540706.1), *Laccaria bicolor* (Lb AMPKp, XP_001884856.1), *Trametes versicolor* (Tv AMPKp, XP_008032462.1), *Dichomitus squalens* (Ds AMPKp, XP_007360734.1), *Parastagonospora nodorum* (Pn AMPKp, XP_001795900.1), *Botrytis cinerea* (Bc AMPKp, XP_001549468.1), *Sclerotinia sclerotiorum* (Ss AMPKp, XP_001588878.1), *Marssonina brunnea* (Mb AMPKp, XP_007292540.1). The horizontal bar indicates the relative distance in the phylogenetic tree.

Figure 5A shows the lysine content of *F. filiformis* under optimal citric acid induction. Organic acids might affect the gene expression of some enzymes in metabolic processes, thereby affecting the whole biosynthesis. The content of lysine in *F. filiformis* under the optimal induction of citric acid, and the gene expression of lysine-biosynthesis-related enzymes, were detected. As shown in Figure 5B, under the optimal induction of citric acid, the content of lysine in *F. filiformis* increased significantly; the gene expression of *hcs*, *hac,* and *hah* was 2.67, 1.97, and 1.90 times greater, respectively, relative to the control, whereas no significant difference was seen for *hdh*, *aat*, *sr*, and *shd*, and the expression of *aar* decreased.

Main energy metabolism and amino acid metabolism regulatory proteins, along with their expression levels, were tested to verify their effects on citric acid regulation of the lysine synthesis pathway. AMPK, an important intracellular energy sensor, is an energy regulatory switch in vivo. GCN2, the only eIF2α kinase in *S. cerevisiae*, was the first one identified as a kinase essential for growth under amino acid starvation conditions [25]. The phosphorylation of eIF2α by GCN2 induces the translation of GCN4 mRNA, encoding the transcriptional activator of amino acid biosynthetic enzymes, while inhibiting the translation of most other messages. Certain amino acids activate the master growth-regulating kinase TOR responding to amino acid starvation, while the increased expression of GCN4 and its amino acid biosynthesis target genes in starved cells is known as general amino acid control (GAAC) [26]. AreA functions as a global nitrogen regulator regulating the utilization of nitrogen sources [27]. As shown in Figure 5C, among genes related to amino acid metabolism and energy metabolism, the expression of the *Ampk* gene was the highest, which was 42.68 times that of the control. Based on the reported *Ampk* nucleotide sequence in *G. lucidum*, the gene fragment of *Ampk* in *F. filiformis* was found by comparison with that in the genome database of *F. filiformis* (Accession: BankIt2661711 Flammulina OQ230329). The amino acid sequence of the protein encoded by the *Ampk* fragment of *F. filiformis* was determined using Basic Local Alignment Search Tool. In order to analyze the evolutionary relationship and kinship between the AMPK proteins in *F. filiformis* and other species, cluster analysis was performed using MEGA.6.0 to compare the AMPK amino acid sequence of *F. filiformis* with the protein sequences reported in other fungi. A total of 12 species were included in the phylogenetic tree of AMPK: ascomycetes and basidiomycetes. The results showed that the *F. filiformis* AMPK protein was more closely related to AMPK homologs in basidiomycetes than homologs in ascomycetes, a result consistent with the species classification of *F. filiformis* (Figure 5E). Expasy predicted the molecular weight of the AMPK protein to be 75.4 kDa and that the theoretical pI would be 4.82, which is similar to the molecular weight and isoelectric point of other known basidiomycete AMPK proteins. After alignment with the other three basidiomycetes, the sequence alignment results showed that the similarity between them was 76.58%. A conserved protein kinase domain (11–265 aa, Accession: cl21453, red box in Figure 5D), a ubiquitin-associated (UBA) domain (309–360 aa, Accession: cl21463, green box in Figure 5D), and an AMPK C-terminal α-subunit regulatory domain (504–670 aa, Accession: cl17070, yellow box in Figure 5D) are present in the AMPK amino acid sequence.

### 3.5. Changes in the Phosphorylation and Lysine Content of Ampk-Silenced Strains under Optimal Induction by Citric Acid

**Figure 6:**
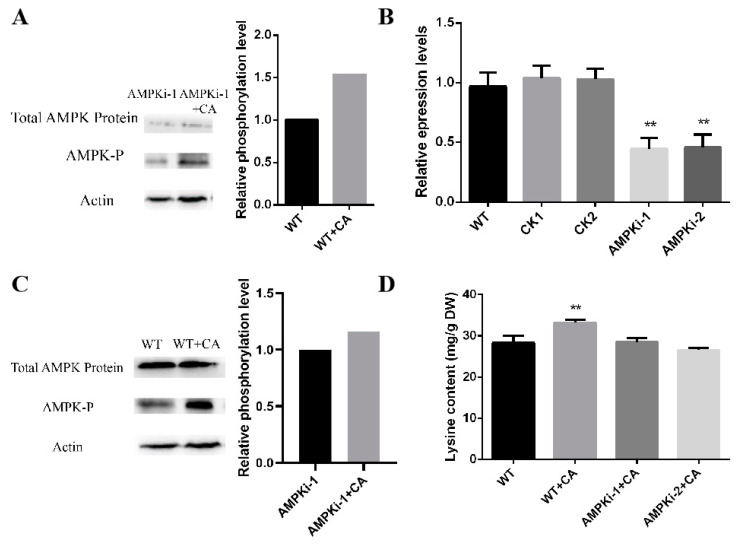
Changes in lysine in AMPK-silencing transformant induced by citric acid. (**A**) AMPK total protein indicates the detection of AMPK total protein level under optimal induction of WT and citric acid; AMPK-P indicates the detection of AMPK phosphorylation level under the optimal induction of WT and citric acid. The level of relative phosphorylation induced by optimal citric acid was measured using Western blot band brightness, and the phosphorylation level of AMPK in WT was set to 1. (**B**) The figure shows the quantitative transformation of silencing transformants by fluorescence quantitative PCR. (** *p* < 0.01). (**C**) The phosphorylation level of AMPK-silenced transformants is shown. AMPK total protein indicates detection of silenced transformant uninduced and citric acid optimal induction of AMPK total protein level. AMPK-P indicates detection of silent transformant uninduced and optimal citric acid induction lower AMPK phosphorylation level. The relative phosphorylation level of the silenced strain under optimal citric acid induction is shown. The brightness of the Western blot band was determined, and the phosphorylation level of AMPK in the silenced strain was set to 1. (**D**) The lysine content of *F. filiformis* AMPK-silencing strain under citrate uninduced and optimal induction. (** *p* < 0.01).

Therefore, we hypothesized that AMPK plays a key role in citrate-induced lysine biosynthesis. The total protein level and phosphorylation level of AMPK were detected with AMPK protein antibody and phosphorylated antibody. The study found that citric acid can significantly increase the phosphorylation level of AMPK in *F. filiformis* and, at the same time, significantly increase the biosynthesis of lysine in *F. filiformis* (Figure 6A). These results suggest that citric acid may affect energy metabolism by increasing AMPK phosphorylation levels and thereby inducing lysine biosynthesis. After the construction of *Ampk*-silenced strains, two silencing transformants (AMPKi-1 and AMPKi-2) with AMPK-silencing efficiencies of about 43% and 47%, respectively, were selected (Figure 6B), and the relative phosphorylation level in *F. filiformis* induced by citric acid was further detected. The results showed a significant decrease in AMPK phosphorylation (Figure 6C). The AMPK phosphorylation level in the AMPK-silencing transformant increased around 16% under optimal citric acid induction, while it increased around 54% in the WT strain under the same treatment. Further citric acid induction found that the lysine content in the AMPK-silencing strains under optimal citric acid induction remained at the same level as in the WT (Figure 6D). Taken together, this shows that AMPK is involved in the regulation of citric-acid-induced lysine biosynthesis in *F. filiformis*.

## 4. Discussion

Various studies have been conducted to increase the lysine content of *F. filiformis* by induction. A proteomic study of the mycelium of *F. filiformis* revealed that only aminoadipate-semialdehyde dehydrogenase in the lysine biosynthesis pathway was upregulated under low-temperature treatment conditions [28]. Induction using chitosan caused an increase in substrate growth and lysine content, and genetic screening revealed a significant increase in the expression of *AAT3* and *AAT4* [16]. However, citric acid not only has the ability to achieve results similar to those of the other inducers, but as a common additive in food it is also cheap, easily available, and highly safe for use in large-scale factory production. Supplementing citric acid under Cr stress increased *CAT*, *POD,* and *GR* activities and metabolites (glutathione and proline) in rice, as well as the restoration of growth parameters, total protein content, and membrane stability [29]. However, few studies apply citric acid as a direct inducer to modulate biosynthesis. As an important component of the microbial metabolic system, citric acid is not only involved in the TCA cycle to provide energy for cellular activities, but also acts as an essential carbon skeleton for various types of biosynthesis. The addition of citric acid promotes energy metabolic processes and provides a substrate for lysine biosynthesis in *F. filiformis*. Therefore, using citric acid for both the induction of lysine biosynthesis and its regulation mechanism in *F. filiformis* would be highly feasible and valuable.

The biosynthesis of lysine by the α-aminoadipate pathway is generated by eight enzymatic reactions that are regulated by the general mechanism for the control of amino acid biosynthesis, as well as by a pathway-specific co-inducer-dependent transcriptional activation. The type strain of *Saccharomyces cerevisiae* and the human pathogenic fungus *Candida* spp. have been used more frequently in studies of the fungal lysine biosynthesis pathway. In *S. cerevisiae*, the α-aminoadipate pathway is initiated by homocitrate synthase, which catalyzes the first and rate-limiting step in the pathway and is highly regulated to conserve the use of resources [17]. Teves et al. found that homocitrate was a regulatory substance controlling the lysine biosynthesis pathway by mutating the homoaconitase of *Penicillium chrysogenum* to achieve a significant accumulation of intracellular homocitrate, which caused a significant increase in the expression of lysine-biosynthesis-related genes, whereas the genes were normally expressed in a lysine auxotroph defective in the homocitrate synthase or in another strain disrupted in the α-aminoadipate reductase [30]. O’Doherty et al. exposed a saccharopine dehydrogenase gene mutant (lys1Δ) strain of *S. cerevisiae* to lipid oxidant-linoleic acid hydroperoxide (LoaOOH) using a medium containing sufficient lysine, finding that it grew significantly slower compared to the wild-type BY4743 strain [31]. Furthermore, despite a full complement of lysine biosynthetic genes, the growth of wild-type BY4743 exposed to LoaOOH was also greatly reduced in lysine-deficient conditions, suggesting that lysine availability and its biosynthesis pathway play an important role in protecting the cell from oxidative stress. Zhou et al. used the natural lysine riboswitch of *E. coli* (ECRS) and *Bacillus subtilis* (BSRS) to control the *gltA* gene and TCA cycling activity in the lysine-producing strain *Corynebacterium glutamicum* LP917 [32]. Both lysine riboswitch-gltA mutants grew more slowly, indicating that TCA cycle activity was reduced. Lysine production was 63% higher in mutant ECRS-gltA and 38% higher in mutant BSRS-gltA, indicating a higher metabolic flux into the lysine biosynthesis pathway. Liu et al. overexpressed the gene *Fvsdh* encoding saccharopine dehydrogenase in *F. filiformis*, which increased the gene expression level by 1.1–3-fold and the lysine content in the transformants by 1.12–1.3-fold [33]. This work found that lysine content increased by nearly 13% under optimal citric acid induction conditions, with the gene expression levels of *hcs*, *hac,* and *hah* being 2.67, 1.97, and 1.90 times higher than the control, respectively. Citric acid induction creates more metabolic flux into lysine biosynthesis, along with upregulating lysine-biosynthesis-related enzymes, and thus significantly increases the lysine content in *F. filiformis*, which is in line with previous studies proving that synthetic substrate accumulation accelerates biosynthesis pathways [30,32].

Our previous study focused on the regulation mechanism of synthetase related to secondary metabolites biosynthesis [34]. In that article, we found that both the lysine levels and growth of *F. filiformis* were significantly increased, which suggests that basal metabolism was also increased; therefore, regulators concerning not only lysine biosynthesis, but also growth and energy metabolism were involved in the process. In studies on *C. albicans*, the transcriptional activator GCN4 was found to be essential for its growth in the presence of lysine deficiency, and GCN4 is a direct activator of several genes in the lysine biosynthetic pathway [35]. As a result, the expression level of these key regulators, *Ampk*, *GCN2*, *GCN4*, *TOR,* and *Area*, were tested, and among them the expression level of *Ampk* was the highest, at 42.68 times higher than that of the control. Thus, we hypothesize that AMPK plays a key role in citric-acid-induced lysine biosynthesis in *F. filiformis*. Adenosine 5’-monophosphate (AMP)-activated protein kinase (AMPK) is a downstream component of a protein kinase cascade that plays a pivotal role in the regulation of intracellular energy status and acts as a measure of cellular energy levels. Acting as an energy sensor in cells, AMPK functions as an energy regulatory switch in organisms to maintain energy stores by regulating anabolic and catabolic pathways [36]. AMPK is highly conserved in eukaryotes and plays a key role in coping with metabolic stress. It has been found that many environmental stress conditions such as hypoxia, heat stress, and oxidative stress can also activate AMPK by generating metabolic stress or other signaling pathways, allowing the body to develop coping mechanisms. In mammals, AMPK is associated with obesity, cancer, diabetes, and longevity [37]. In plants, AMPK is involved in growth, development, and morphogenesis [38]. In filamentous fungi, AMPK is involved in mycelium and cell wall formation [39]. Glsnf1, a sucrose-nonfermenting serine-threonine-protein kinase 1 (Snf1)/AMP-activated protein kinase homologue in *Ganoderma lingzhi* [40], actively regulates cellulose-degrading enzymes transcription and activity, and it plays a key role in metabolic remodeling in response to environmental stress [41,42]. Although no direct link between lysine biosynthesis and AMPK has been found, AMPK is an important regulator of energy metabolism, and lysine biosynthesis is closely related to energy metabolism in *F. filiformis*, and an increase in lysine biosynthesis is accompanied by an increase in AMPK phosphorylation levels, so it has been concluded that AMPK plays an important role in the induction of lysine in response to citric acid.

**Figure 7:**
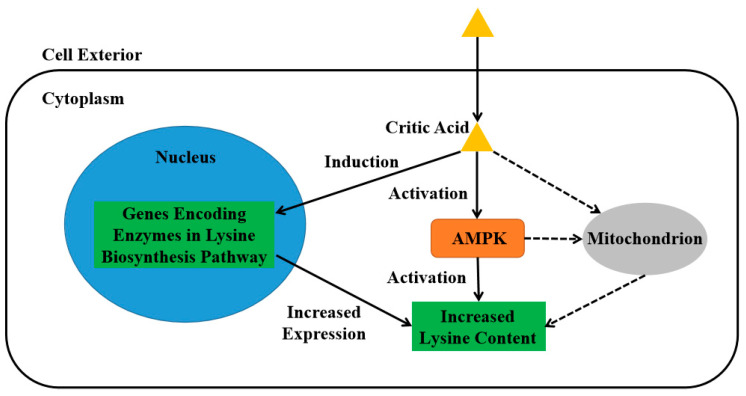
Effects of citric acid induction on lysine biosynthesis in *Flammulina filiformis*. Citric acid treatment induces the upregulation of expression of *AMPK* and genes encoding enzymes in the lysine biosynthesis pathway. AMPK is further activated by the increase in phosphorylation level. Both the upregulation of synthase genes and the activation of AMPK together increase the lysine content in *F. filiformis*. Dotted lines represent the hypothetical series of events not involved in this work.

In summary, it was found that citric acid induction was able to increase biomass and mycelium growth and significantly accelerate lysine biosynthesis in *F. filiformis* mycelium. Citric acid treatment response surface methodology found that the optimal citric acid induction time and concentration were 33.18 h and 138.26 μM, with the theoretical lysine content obtained being 33.92 mg/g DW. The data obtained from the actual measurements were 32.4378, 33.45, and 33.78 mg/g DW, which were in line with the theoretical results. Induction during fruiting body growth found that 0.36 L 1 mM citric acid treatment for 3 days was able to increase the highest level of lysine content by 12.86%. Under the optimal induction of citric acid, biosynthesis-related genes’ expression levels, along with AMPK expression and phosphorylation level, increased as well. Then, AMPK-silencing strains were constructed to block AMPK signaling, resulting in the disappearance of the increased lysine biosynthesis after citric acid induction. It was demonstrated that AMPK is involved in the citric acid regulation of lysine biosynthesis in *F. filiformis* (Figure 7).

## Data Availability

The data presented in this study are available in the article and the Supplementary Materials.

## Supplementary Materials

The following supporting information can be downloaded at: https://www.mdpi.com/article/10.3390/jof9030340/s1, Table S1 Citric acid treatment time and concentration factor level; Table S2 Design matrix of CCD and response; Table S3 Analysis of variance in response surface experiment; Table S4 Primers used.

## References

[1] Dai Y.C., Yang Z.L., Cui B.K., Wu G., Yuan H.S., Zhou L.W., He S.H., Ge Z.W., Wu F., Wei Y.L. (2021). Diversity and systematics of the important macrofungi in Chinese forests. Mycosystema.

[2] Cong W.R., Liu Y., Li Q.Z., Zhou X.W. (2014). Cloning and analysis of a functional promoter of fungal immunomodulatory protein from *Flammulina velutipes*. Mol. Biol. Rep..

[3] Shi C., Wu Y.Y., Fang D.L., Pei F., Mariga A.M., Yang W.J., Hu Q.H. (2018). Effect of nanocomposite packaging on postharvest senescence of *Flammulina velutipes*. Food Chem..

[4] Li H., Shi L., Tang W., Xia W., Zhong Y., Xu X., Xie B., Tao Y. (2022). Comprehensive Genetic Analysis of Monokaryon and Dikaryon Populations Provides Insight into Cross-Breeding of *Flammulina filiformis*. Front. Microbiol..

[5] Liu X., Dong J., Liao J., Tian L., Qiu H., Wu T., Ge F., Zhu J., Shi L., Jiang A. (2022). Establishment of CRISPR/Cas9 Genome-Editing System Based on Dual sgRNAs in *Flammulina filiformis*. J. Fungi.

[6] Ribeiro B., Andrade P.B., Silva B.M., Baptista P., Seabra R.M., Valentao P. (2008). Comparative Study on Free Amino Acid Composition of Wild Edible Mushroom Species. J. Agric. Food Chem..

[7] Smiderle F.R., Carbonero E.R., Sassaki G.L., Gorin P.A.J., Iacomini M. (2008). Characterization of a heterogalactan: Some nutritional values of the edible mushroom *Flammulina velutipes*. Food Chem..

[8] Cai H.H., Liu X.M., Chen Z.Y., Liao S.T., Zou Y.X. (2013). Isolation, purification and identification of nine chemical compounds from *Flammulina velutipes* fruiting bodies. Food Chem..

[9] Irla M., Wendisch V.F. (2022). Efficient cell factories for the production of N-methylated amino acids and for methanol-based amino acid production. Microb. Biotechnol..

[10] Lou H.H., Li H., Wei T.Y., Chen Q.H. (2021). Stimulatory Effects of Oleci Acid and Fungal Elicitor on Betulinic Acid Production by Submerged Cultivation of Medicinal Mushroom Inonotus obliquus. J. Fungi.

[11] Liu Z.C., Liu R.P., Tong X.Y., Zou L. (2022). New Insights into Methyl Jasmonate Regulation of Triterpenoid Biosynthesis in Medicinal Fungal Species Sanghuangporus baumii (Pilat) LW Zhou & YC Dai. J. Fungi.

[12] Dai Y.C., Yang Z.L. (2018). Notes on the nomenclature of five important edible fungi in China. Mycosystema.

[13] Fu Y.P., Liang Y., Dai Y.T., Yang C.T., Duan M.Z., Zhang Z., Hu S.N., Zhang Z.W., Li Y. (2016). De Novo Sequencing and Transcriptome Analysis of *Pleurotus eryngii* subsp. *tuoliensis* (Bailinggu) Mycelia in Response to Cold Stimulation. Molecules.

[14] Xu Y.N., Xia X.X., Zhong J.J. (2013). Induced effect of Na plus on ganoderic acid biosynthesis in static liquid culture of Ganoderma lucidum via calcineurin signal transduction. Biotechnol. Bioeng..

[15] Xu Y.N., Xia X.X., Zhong J.J. (2014). Induction of Ganoderic Acid Biosynthesis by Mn2+ in Static Liquid Cultivation of Ganoderma Lucidum. Biotechnol. Bioeng..

[16] Tian L., Ma Z., Qiu H., Liu X., Wu T., Ge F., Liu R., Zhu J., Shi L., Jiang A. (2022). Chitosan Increases Lysine Content through Amino Acid Transporters in *Flammulina filiformis*. Foods.

[17] Xu H.Y., Andi B., Qian J.H., West A.H., Cook P.F. (2006). The alpha-aminoadipate pathway for lysine biosynthesis in fungi. Cell Biochem. Biophys..

[18] Zabriskie T.M., Jackson M.D. (2000). Lysine biosynthesis and metabolism in fungi. Nat. Prod. Rep..

[19] Lopez-Bucio J., Nieto-Jacobo M.F., Ramirez-Rodriguez V., Herrera-Estrella L. (2000). Organic acid metabolism in plants: From adaptive physiology to transgenic varieties for cultivation in extreme soils. Plant Sci..

[20] Gong M., Huang T.Y., Li Y., Li J.X., Tang L.H., Su E.Z., Zou G., Bao D.P. (2022). Multi-Omics Analysis of Low-Temperature Fruiting Highlights the Promising Cultivation Application of the Nutrients Accumulation in *Hypsizygus marmoreus*. J. Fungi.

[21] Wu T.J., Hu C.C., Xie B.G., Zhang L., Yan S.J., Wang W., Tao Y.X., Li S.J. (2019). A Single Transcription Factor (PDD1) Determines Development and Yield of Winter Mushroom (*Flammulina velutipes*). Appl. Environ. Microbiol..

[22] Wu T., Xia J., Ge F., Qiu H., Tian L., Liu X., Liu R., Jiang A., Zhu J., Shi L. (2022). Target of Rapamycin Mediated Ornithine Decarboxylase Antizyme Modulate Intracellular Putrescine and Ganoderic Acid Content in *Ganoderma lucidum*. Microbiol. Spectr..

[23] Livak K.J., Schmittgen T.D. (2001). Analysis of relative gene expression data using real-time quantitative PCR and the 2(T)(-Delta Delta C) method. Methods.

[24] Zhang G., Sun Z.H., Ren A., Shi L., Shi D.K., Li X.B., Zhao M.W. (2017). The mitogen-activated protein kinase GlSlt2 regulates fungal growth, fruiting body development, cell wall integrity, oxidative stress and ganoderic acid biosynthesis in Ganoderma lucidum. Fungal Genet. Biol..

[25] Sattlegger E., Swanson M.J., Ashcraft E.A., Jennings J.L., Fekete R.A., Link A.J., Hinnebusch A.G. (2004). YIH1 is an actin-binding protein that inhibits protein kinase GCN2 and impairs general amino acid control when overexpressed. J. Biol. Chem..

[26] Gallinetti J., Harputlugil E., Mitchell J.R. (2013). Amino acid sensing in dietary-restriction-mediated longevity: Roles of signal-transducing kinases GCN2 and TOR. Biochem. J..

[27] Horst R.J., Zeh C., Saur A., Sonnewald S., Sonnewald U., Voll L.M. (2012). The Ustilago maydis Nit2 Homolog Regulates Nitrogen Utilization and Is Required for Efficient Induction of Filamentous Growth. Eukaryot. Cell.

[28] Liu J.Y., Chang M.C., Meng J.L., Feng C.P., Wang Y. (2018). A Comparative Proteome Approach Reveals Metabolic Changes Associated with *Flammulina velutipes* Mycelia in Response to Cold and Light Stress. J. Agric. Food Chem..

[29] Khatun M.R., Mukta R.H., Islam M.A., Huda A. (2019). Insight into citric acid-induced chromium detoxification in rice (Oryza sativa. L). Int. J. Phytoremediation.

[30] Teves F., Lamas-Maceiras M., Garcia-Estrada C., Casqueiro J., Naranjo L., Ullan R.V., Scervino J.M., Wu X.B., Velasco-Conde T., Martin J.F. (2009). Transcriptional upregulation of four genes of the lysine biosynthetic pathway by homocitrate accumulation in Penicillium chrysogenum: Homocitrate as a sensor of lysine-pathway distress. Microbiology-Sgm.

[31] O’Doherty P.J., Lyons V., Tun N.M., Rogers P.J., Bailey T.D., Wu M.J. (2014). Transcriptomic and biochemical evidence for the role of lysine biosynthesis against linoleic acid hydroperoxide-induced stress in Saccharomyces cerevisiae. Free. Radic. Res..

[32] Zhou L.B., Zeng A.P. (2015). Exploring Lysine Riboswitch for Metabolic Flux Control and Improvement of L-Lysine Synthesis in *Corynebacterium glutamicum*. ACS Synth. Biol..

[33] Liu J.Y., Li Q.Z., Jiang P.Y., Xu Z., Zhang D., Zhang L.J., Zhang M.Y., Yu H.L., Song C.Y., Tan Q. (2019). Overexpression of the saccharopine dehydrogenase gene improves lysine biosynthesis in *Flammulina velutipes*. J. Basic Microbiol..

[34] Wang Y.H., Yang Z.Y., Chen X., Han D., Han J., Wang L.S., Ren A., Yu H.S., Zhao M.W. (2021). Lenthionine, a Key Flavor Substance in Lentinula edodes, Is Regulated by Cysteine under Drought Stress. J. Agric. Food Chem..

[35] Priyadarshini Y., Natarajan K. (2016). Reconfiguration of Transcriptional Control of Lysine Biosynthesis in Candida albicans Involves a Central Role for the Gcn4 Transcriptional Activator. Msphere.

[36] Woods A., Dickerson K., Heath R., Hong S.P., Momcilovic M., Johnstone S.R., Carlson M., Carling D. (2005). Ca^2+^/calmodulin-dependent protein kinase kinase-beta acts upstream of AMP-activated protein kinase in mammalian cells. Cell Metab..

[37] Xiao B., Sanders M.J., Underwood E., Heath R., Mayer F.V., Carmena D., Jing C., Walker P.A., Eccleston J.F., Haire L.F. (2011). Structure of mammalian AMPK and its regulation by ADP. Nature.

[38] Baena-Gonzalez E., Rolland F., Thevelein J.M., Sheen J. (2007). A central integrator of transcription networks in plant stress and energy signalling. Nature.

[39] Backhaus K., Rippert D., Heilmann C.J., Sorgo A.G., de Koster C.G., Klis F.M., Rodicio R., Heinisch J.J. (2013). Mutations in SNF1 complex genes affect yeast cell wall strength. Eur. J. Cell Biol..

[40] Dai Y.C., Cao Y., Zhou L.W., Wu S.H. (2013). Notes on the nomenclature of the most widely cultivated Ganoderma species in China. Mycosystema.

[41] Hu Y.R., Xu W.Z., Hu S.S., Lian L.D., Zhu J., Shi L., Ren A., Zhao M.W. (2020). In Ganoderma lucidum, Glsnf1 regulates cellulose degradation by inhibiting GlCreA during the utilization of cellulose. Environ. Microbiol..

[42] Hu Y.R., Xu W.Z., Hu S.S., Lian L.D., Zhu J., Ren A., Shi L., Zhao M.W. (2020). Glsnf1-mediated metabolic rearrangement participates in coping with heat stress and influencing secondary metabolism in Ganoderma lucidum. Free. Radic. Biol. Med..

