# Supplementary material for: The AMP-Activated Protein Kinase (AMPK) Positively Regulates Lysine Biosynthesis Induced by Citric Acid in Flammulina filiformis
Source: J Fungi (Basel). 2023 Mar 10;9(3):340. doi: 10.3390/jof9030340 (PMC10057554; doi:10.3390/jof9030340)
Supplement: Supplementary file 1 [file jof-09-00340-s001.zip › jof-2229051-supplementary.pdf]

Supporting information

Table S1 Citric acid treatment time and concentration factor level

Factor	Name	Level	Low level	High Level
A	Time	36.00	7.83	64.17
B	Concentration	150.00	32.62	267.38

Table S2 Design matrix of CCD and response

Run	Factor 1<br>A:Time(h)	Factor 2<br>B:Dose(μM)	Response 1<br>Total flavonoids(mg/mL)
1	0.00	150.00	25.34
2	64.17	267.38	23.12
3	7.83	267.38	23.43
4	72.00	150.00	27.14
5	64.17	267.38	23.39
6	7.83	267.38	23.81
7	0.00	150.00	26.53
8	64.17	32.62	22.28
9	36.00	150.00	32.26
10	0.00	150.00	26.45
11	7.83	32.62	24.58
12	36.00	0.00	26.11
13	36.00	300.00	26.07
14	7.83	32.62	22.24
15	72.00	150.00	26.64
16	64.17	32.62	21.48
17	36.00	0.00	26.83
18	72.00	150.00	26.91
19	36.00	0.00	26.53
20	36.00	300.00	25.99
21	36.00	300.00	26.11

A five-level-two-factor design requiring 21 experimental combinations was employed in this optimization study. Each experiment was repeated three times and the mean Lysine value was calculated. The experimental data was analyzed by a second order polynomial regression.

Table S3 Analysis of variance in response surface experiment

Source	Sum of Squares	df	Mean Square	F Value	P-value Prob>F	
Model	90.60	5	18.12	12.26	< 0.0001	significant
A-time	0.032	1	0.032	0.022	0.8847	
B-Concentration	0.13	1	0.13	0.087	0.7721	
AB	0.68	1	0.68	0.46	0.5078	
A^2	78.13	1	78.13	52.88	< 0.0001	
B^2	82.70	1	82.70	55.97	< 0.0001	
Residual	22.16	15	1.48			
Lack of Fit	17.73	3	5.91	16.02	0.0002	significant
Pure Error	4.43	12	0.37	12.26	< 0.0001	
Cor Total	112.76	20	18.12			

Table S4 Primers used

Primer	Sequence
D-HCS-F	TCACACACAAGGCAGGTATTC
D-HCS-R	CATATCGCGTGAGACCAAAGT
D-HCD-F	TCCAAGGCTGGTGCTATTG
D-HCD-R	CCGGATACAAACATGGGTATGA
D-HAH-F	CGCCCTCTTTGGTTCAGTTA
D-HAH-R	TCGGACATTCCCGTAGAGAT

D-AAT-F	CACAGGGTGCACTATGTCTAAC
D-AAT-R	GCTTCTGACCGAGAAGGTATTG
D-AAR-F	GACGGTGGATGACGAGATAATG
D-AAR-R	CCAGCTGCAAGAGTAGGTAAAG
D-SR-F	GAGATCCTGAGTGGCCATTATC
D-SR-R	GGTATCTTCCGTGGTGGAATAG
D-SDH-F	GGCTTCAGATTCAGGCTATCA
D-SDH-R	GAGTGGAGTCACCAGTCTTTC
D-NR-F	TGATTGGGTAAAGCGGGATAA
D-NR-R	GAGTAAGGAAGCCTGCAGTATAG
D-AMPK-F	TATGGAGCTGTGGCGTAATC
D-AMPK-R	GCAGACAGATGGTGAGGTATAG
D-GCN4-F	TCGTATCCCTCTTCTGCTCTTC
D-GCN4-R	GGTTGGGTAGGTTCATCCATTC
D-GCN2-F	GCAAGAGTTCGATGACCAGATA
D-GCN2-R	ACTTCGGTTGACTCGGAATTAG
D-AREA-F	CCAGACTACGAACACTCCTCTA
D-AREA-R	TCTTCAGCGACAAAGGTCTTAC
D-TOR-F	AAGTTGTATTCTCGCGTCCTC
D-TOR-R	GTCGGTCTGGCATGATTGTA
LYZ-NRi-F	ACTGggtaccCTTGGACTCTGGCTGATA
LYZ-NRi-R	ACTGactagtAACGGGCTTTAGGATGT

LYZ-AMPKi-F	ACTGggtaccGCGACAAGACGAAACCG
LYZ-AMPKi-R	ACTGactagtGGCCACCACGTATAACCT
HPH-F	GTGCTTGACATTGGGG
HPH-R	TTCGGGGCAGTCCTCG

Ampk* sequence of *F. fifiliformis

ATGTCGGCAACTGTGTTTCCAGCGTCCAAGCTTGGAGAGTATCTTGTCACTGAGGACATTG
CAGAAGGCACGTTCGGCATCGTCAAAAAGGCCATACACACCATTACTGGCCATGAAGTCGC
TCTCAAATACATCTCCAAGGCTGCGATTCACCGCGACAAGACGAAACCGCGCGTTCGCCGC
GAGTTCGAGTACATGCGCACGCTCAGACACCCACATATCATTAAACTCTACGAAGTCATTTC
TACGCCGTCGGACATTATCTTTGTACTCGAATATGCCAAAGGCGAGCTCTTCAACTACATCG
TCCAGACTGGCTACATGGAGGAAGACCAAGCCCGCCGCTTCTTCCAGCAAATTATCTCCGG
CATGGAATACTCGCACCGGCTAAAAATCGTCCATCGCGATCTGAAGCCAGAAAATATCCTTC
TCGACGACAACCTCAACGTCAAGATCACAGACTTTGGGCTCTCCAGCGAGATATCGGATGG
CTCCTTCCTCACCACCAGTTGCGGAAGCCCCAACTACGCAGCCCCGGAGGTTATACGTGGT
GGCCAGTATGCCGGTCCCGAGATAGACGTATGGAGCTGTGGCGTAATCCTCTACGTGATGCT
CTCTGGCAGACTGCCCTTTGACGACGCCGACATGAACACCCTCTTCCACAAAATAGGCCAT
GGCCATTTTACTATACCTCACCATCTGTCTGCCGACGCCACTGATCTCATCAGACGCATGCTA
GTCGTTGATCCCTTAGATCGCATAACAATTCCAGAAATCACTCAACATCCCTTCTTCACGAA
AAATTTGGCCCGCTACCTTTCCCCTCTTCCTCCGCCGCCTGGCCCAGTTCTCGGCACCCTCT
CCTCCCTCGTCACACAGCCACAACACAAGCTCGACTTTGAGTATGTCGAGGGCCTGGGTCC
CGTCGAAGACAGCCTCGTTGAGGAACTCGCTTCTCGTCTGGAGGGTATCACCAAGGACGA

CGTCTGGGACAGTCTTCGACGCAATGACGGTGTCCAAGGAAACTCTGTCAAGGTTGCCTAC
CAGCTACTCAAGGACAAGAAACACCTGGGCAAGGATCTCGCTCGCTATGCTGATCACGAGC
GTGATGTGCAGGAGGCCGCATTAGATGTAACTACTCTTCATTCTTACTTGCGCATCGCCATCG
CTTACCTTCAGACTGTCCAGCCGCGCAACGCAGTATCGCCACAGGCTCGGTCTCCAGGTGG
TCGCGACCTCGATGATAACCCCTTCGAAGAAGCTTTCGGAGCTGGTGGACCTTCCGTCGAA
GATGAACTGGGATTAGATTCGGAGCCAGACACTTCGGACGACTCCGACGAAGAATACGATT
CACAAGAAGAAGAGGAGGAGCCTATAGAGCCCTCCACCTCCGAAACCTTTGCCGTCCTCA
ACTCCTCCCTTCCCGAAAAACTCGTCGCCTCTCCCTCCTCACACCACCTCGCGTCGTACGC
AACGGCACGGCGCTCCGCAGGGCAAAAGAAACAACACCGTTCGAAGTGGCACTTTGGTAT
ACGCAGCCGGAGTGCGCCGTTTGAGATTATTCTGGAAGTGTATCGTGTTCTGCAGGATTTGG
GGATGGAGTGGAAGACGAGGCCGGGTCCGAAGGGGCTGAGGAAGAATGCGTCGCATGGC
GGAGGATCGATCGCGCTGGCGAGTGAATTTGATGGTGGCGGGCGGGTCGACCTCAAGTCG
CTTTTTATGATTGAGAGCCGCGCGAGGATGCGGGATGTTGTGGTGCTGATGGATCTGAGGTT
GTACAATATCGACAAGGTCCATTATCTCGTGGACTTTCACCATAAAAAGACGTACCGCGCGT
CTACGCTTCCCGGGGCGGGGAAATTTGATATGGCGCCTGCTATTTCACCGACTGCGTCGTTC
TCGGCGTTGTCTGACTCTGCGCGGGCGGCGACTGTCACGAGTCGGGAGGATGATATCGTGG
TCTCTCCGTATACGTTTATGGATGTGGCGTGTAACATCATTCTTGTGTTGGCGAGTGGGAAC
GTGGAGCCATGA

AMPK amino acid sequence of *F. fifiliformis*

MSATVFPASKLGEYLVTEDIAEGTFGIVKKAIHTITGHEVALKYISKAAIHRDKTKPRVRREFEY
MRTLRHPHIIKLYEVISTPSDIIFVLEYAKGELFNYIVQTGYMEEDQARRFFQQIISGMEYSHRLK

IVHRDLKPENILLDDNLNVKITDFGLSSEISDGSFLTTSCGSPNYAAPEVIRGGQYAGPEIDVWSC
GVILYVMLSGRLPFDDADMNTLFHKIGHGHFTIPHHLSADATDLIRRMLVVDPLDRITIPEITQH
PFFTKNLARYLSPLPPPPGPVLGTLSSLVTQPQHKLDFEYVEGLGPVEDSLVEELASRLEGITKD
DVWDSLRRNDGVQGNSVKVAYQLLKDKKHLGKDLARYADHERDVQEAALDVTTLHSYLRIA
IAYLQTVQPRNAVSPQARSPGGRDLDDNPFEEAFGAGGPSVEDELGLDSEPDTSDDSDEEYDSQ
EEEEEPIEPSTSETFAVLNSSLPEKLVASPSSHHLASYATARRSAGQKKQHRSKWHFGIRSRSAPF
EIILEVYRVLQDLGMEWKTRPGPKGLRKNASHGGGSIALASEFDGGGRVDLKSLFMIESRARM
RDVVVLMDLRLYNIDKVHYLVDFHHKKTYRASTLPGAGKFDMAPAISPTASFSALSDSARAAT
VTSREDDIVVSPYTFMDVACNIILVLASGNVEP
